# Investigation of the Corrosion and Wear Behavior of Electrochemically Deposited Zn-Co-Graphene-TiO_2_ Nanocomposite Coatings on Ti6Al4V Substrates Fabricated by Selective Laser Melting (SLM)

**DOI:** 10.3390/ma19173784

**Published:** 2026-09-05

**Authors:** Mustafa Yazici

**Affiliations:** Department of Basic Sciences, Faculty of Science, Erzurum Technical University, Erzurum 25050, Türkiye; mustafa.yazici@erzurum.edu.tr

**Keywords:** selective laser melting, graphene, TiO_2_, electrodeposition, Ti6Al4V

## Abstract

This study investigates the microstructural, tribological, and corrosion properties of electrodeposited Zn-Co nanocomposite coatings reinforced with graphene and TiO_2_ nanoparticles on Selective Laser-Melted (SLM) Ti6Al4V alloy. Systematic characterization using XRD, SEM, and Raman spectroscopy revealed that the incorporation of graphene and TiO_2_ significantly refined the grain structure, resulting in a dense and defect-free surface morphology. Reciprocating wear tests demonstrated that the optimized hybrid coating (Zn-Co-GTi) exhibited superior tribological performance. Electrochemical impedance spectroscopy (EIS) tests conducted in simulated body fluid (SBF) at 37 °C demonstrated that the optimized hybrid coating (Zn-Co-GTi) also provided enhanced corrosion resistance. Specifically, the coefficient of friction decreased from 0.79 to 0.24, while the wear rate was reduced to 5.1 × 10^−4^ mm^3^/Nm. Electrochemical evaluations further confirmed a significant improvement in corrosion resistance, with the Zn-Co-GTi coating exhibiting the lowest corrosion current density (0.0059 μA cm^−2^) and the highest charge transfer resistance (Rct). However, increasing the reinforcement content beyond the optimum level resulted in partial nanoparticle agglomeration, leading to a slight deterioration in both tribological and corrosion performance. Overall, the optimized Zn-Co-Graphene-TiO_2_ nanocomposite coating provides an effective and scalable surface engineering strategy for improving the durability and corrosion resistance of SLM-produced Ti6Al4V components for advanced engineering and biomedical applications.

## 1. Introduction

Titanium and its alloys are widely recognized as high-performance engineering materials and have been extensively employed in a variety of industrial and biomedical applications due to their exceptional combination of properties, including high specific strength, excellent corrosion resistance, superior biocompatibility, relatively low density, and good mechanical toughness [1]. Among titanium alloys, Ti6Al4V has received particular attention in biomedical engineering, especially in dental and orthopedic applications, owing to its favorable mechanical characteristics and excellent compatibility with biological tissues. These properties make Ti6Al4V a preferred material for the fabrication of implants, prosthetic devices, and bone fixation components. Despite these advantages, the fabrication of titanium components with complex geometries using conventional manufacturing techniques remains challenging. Titanium alloys typically exhibit poor machinability because of their high chemical reactivity, low thermal conductivity, and strong tendency for tool wear during processing. In recent years, additive manufacturing technologies have emerged as promising alternatives to overcome these limitations. In particular, selective laser melting (SLM) has gained considerable attention due to rapid advancements in powder feedstock development, process control systems, and digital design tools. SLM enables the layer-by-layer fabrication of complex structures with minimal geometric constraints, allowing the production of customized components that would be difficult or impossible to achieve using traditional manufacturing approaches [2]. Consequently, the production of patient-specific Ti6Al4V implants with intricate geometries has become increasingly feasible and widely adopted in biomedical engineering [3,4,5].

However, despite their favorable bulk properties, SLM-fabricated titanium components exhibit intrinsic surface limitations, such as high surface roughness, poor wear resistance, and a high coefficient of friction, which can lead to severe tribological degradation, debris formation, and accelerated localized corrosion in biological environments [6,7]. To address these challenges, various surface modification and protective coating strategies have recently been explored to enhance the tribological and electrochemical stability of Ti6Al4V alloys without compromising their bulk mechanical integrity. Among these, considerable research efforts have focused on improving the surface properties of titanium alloys through various surface engineering strategies. Among these approaches, surface modification and coating technologies have emerged as effective methods for enhancing wear resistance, reducing friction, and improving corrosion and tribological performance without compromising the desirable bulk properties of the substrate material.

In particular, composite coatings have gained significant attention because they combine the advantageous properties of different materials within a single coating system. By incorporating reinforcing particles into a metallic matrix, composite coatings can markedly improve hardness, wear resistance, and load-bearing capacity while also reducing friction and enhancing corrosion resistance. Moreover, the ability to tailor the type, size, and distribution of the reinforcing phases allows the surface properties to be optimized for specific applications, making composite coatings an effective strategy for enhancing the functional performance of titanium-based materials. In recent years, the growing demand for advanced materials with superior mechanical, tribological, and corrosion-resistant properties has driven significant research interest in composite coatings based on zinc and its alloys. Among these, Zn–Co alloy coatings have emerged as promising alternatives to traditional electroplated systems such as zinc or cobalt, due to their excellent corrosion resistance, environmental compatibility, and cost-effectiveness. The synergistic interaction between zinc and cobalt enhances both the cathodic protection capability of zinc and the structural stability imparted by cobalt, resulting in coatings that demonstrate improved hardness, adhesion, and durability in aggressive environments [8]. Despite these advantages, conventional Zn–Co coatings often face challenges in achieving high mechanical integrity and long-term corrosion resistance under harsh operating conditions [9].

The incorporation of ceramic and carbon-based nanoparticles into the metallic matrix has been demonstrated to be a highly effective strategy for overcoming these challenges. In this context, the co-deposition of nanomaterials such as graphene and titanium dioxide (TiO_2_) within the Zn–Co matrix has attracted substantial attention. Among hybrid reinforcements, graphene and TiO_2_ nanoparticles have been widely investigated [10,11,12]. These hybrid reinforcements can significantly enhance coating performance by refining the grain structure, improving barrier properties, and facilitating uniform current distribution during the electrodeposition process [13]. Graphene, the first of these materials, consists of a two-dimensional sheet of sp^2^-hybridized carbon atoms arranged in a honeycomb lattice and demonstrates exceptional electrical conductivity, mechanical strength, and chemical stability [14,15,16]. When incorporated into metal matrices, graphene nanosheets act as a conductive scaffold that promotes uniform nucleation and growth of the metal grains. Furthermore, the impermeable nature of graphene provides an effective barrier against corrosive ions such as chloride, thereby reducing the rate of electrochemical degradation [17,18,19]. In Zn-Co systems, graphene addition has been shown to improve microhardness, wear resistance, and corrosion protection due to its synergistic interaction with cobalt and zinc ions during deposition [16].

Another hybrid additive is TiO_2_ nanoparticles, which are chemically stable and non-toxic ceramic nanoparticles known for their excellent photocatalytic and passivation properties [20,21]. TiO_2_ reinforcement enhances the microstructural compactness of the coating, leading to the formation of finer grains and a reduction in porosity [22]. Additionally, TiO_2_ nanoparticles improve the hydrophobicity and passivation behavior of the composite, thereby minimizing corrosive attack [23]. When TiO_2_ and graphene are simultaneously incorporated into Zn-Co coatings, they often exhibit a synergistic effect, where graphene provides a conductive network facilitating charge transfer, while TiO_2_ contributes to physical barrier strengthening and chemical passivation [24]. This hybrid reinforcement approach enables the fabrication of multifunctional coatings with superior performance compared to those reinforced with a single type of nanoparticle. The presence of graphene and TiO_2_ nanoparticles in the plating bath modifies the local current distribution and nucleation kinetics, promoting the formation of compact and uniform coatings. However, challenges such as particle agglomeration and poor dispersion stability in the electrolyte must be carefully addressed to ensure homogeneous incorporation of nanoparticles. The use of surfactants or mechanical agitation can mitigate these issues, leading to improved dispersion and interfacial bonding between the matrix and reinforcement phases [25,26].

Recent studies have demonstrated that graphene and TiO_2_ co-deposition in Zn-based systems results in remarkable improvements in both mechanical and electrochemical performance. For example, the inclusion of graphene has been reported to increase microhardness by up to 40%, while TiO_2_ enhances the corrosion resistance by forming a dense passive layer on the surface [27]. Furthermore, the synergistic addition of both reinforcements can significantly reduce the corrosion current density and improve the charge transfer resistance in electrochemical impedance spectroscopy (EIS) analyses. These enhancements are primarily attributed to the refined grain structure, reduced surface defects, and improved interfacial cohesion between the reinforcement and the metal matrix [28]. In addition to corrosion protection, wear resistance is another critical parameter for evaluating the functional performance of Zn–Co–graphene–TiO_2_ composite coatings. The incorporation of hard TiO_2_ nanoparticles acts as a solid lubricant and load-bearing constituent, reducing the coefficient of friction and material loss during sliding contact [29]. Meanwhile, the presence of graphene provides an additional lubricating layer, minimizing wear-induced surface degradation. This dual reinforcement mechanism contributes to a substantial improvement in tribological behavior, making these coatings suitable for applications involving high friction and wear, such as mechanical joints, turbine blades, and biomedical implants [30]. Another important consideration is the interaction between graphene and TiO_2_ within the coating matrix. Graphene can act as an electron mediator for TiO_2_, enhancing its photocatalytic and charge transfer capabilities, while TiO_2_ can prevent graphene restacking, thereby preserving its high surface area and mechanical integrity. This mutual reinforcement effect contributes to improved coating compactness, electrical conductivity, and overall stability. Such synergistic mechanisms have been explored in various studies, revealing that hybrid coatings exhibit up to an order of magnitude improvement in corrosion resistance compared to monolithic Zn-Co coatings. The application of these advanced composite coatings on Ti6Al4V substrates aligns well with current trends in surface engineering, where the goal is to integrate the desirable properties of multiple materials into a single functional layer. The ability to deposit Zn-Co-G-TiO_2_ coatings via electrodeposition provides a cost-effective, scalable, and environmentally friendly pathway for enhancing the surface performance of critical components.

In summary, the development of Zn-Co-based composite coatings reinforced with graphene and TiO_2_ nanoparticles represents a significant advancement in the field of protective surface engineering. The combined benefits of graphene’s exceptional conductivity and TiO_2_’s robust chemical stability create a multifunctional hybrid coating capable of resisting corrosion, wear, and mechanical degradation. When applied to Ti6Al4V substrates, such coatings have the potential to overcome the intrinsic limitations of titanium alloys and extend their operational lifetime under challenging conditions.

Although several studies have investigated binary electrodeposited systems containing Graphene or TiO_2_ individually, a major research gap remains regarding the synergistic incorporation of hybrid Graphene and TiO_2_ reinforcements into electrodeposited Zn–Co matrices directly applied to 3D-printed (SLM) Ti6Al4V substrates. The unique surface morphology of SLM substrates presents distinct nucleation and growth dynamics during electrodeposition compared to conventional wrought titanium. Therefore, the novel objective of the present study is to systematically synthesize and optimize electrodeposited Zn–Co–Graphene–TiO_2_ nanocomposite coatings on SLM Ti6Al4V alloys. This work provides a quantitative composition–microstructure–performance relationship, elucidating the dual lubrication and corrosion barrier mechanisms, and identifying the precise nanoparticle threshold beyond which agglomeration occurs in simulated body fluid (SBF) environments.

## 2. Experimental Details

### 2.1. Substrate Preparation (Ti6Al4V)

In this study, Ti6Al4V alloy (Grade 23) substrates were fabricated using a Concept Laser MLab Cusing R (Concept Laser GmbH, Lichtenfels, Germany) selective laser melting (SLM) system equipped with a 100 W continuous-wave Yb-fiber laser (1070 nm wavelength, spot diameter of ≈50 µm). To ensure high reproducibility, the processing parameters recommended by the equipment manufacturer for Ti6Al4V processing were applied as follows:

- Laser power: 90 W
- Scanning speed: 600 mm/s
- Hatch spacing: 60 µm
- Layer thickness: 25 µm
- Scanning strategy: Island scanning pattern with a 45° rotation between successive layers to minimize residual stress accumulation.
- Build orientation: Flat horizontal (0°) relative to the build platform.

All specimens were fabricated under a strictly controlled inert argon atmosphere to keep the oxygen content below 0.1%. Following fabrication, a stress-relief heat treatment was performed at 650 °C for 2 h in a vacuum furnace, followed by slow furnace cooling to eliminate residual thermal stresses induced during laser scanning. The relative density of the as-built SLM substrates was measured to be 99.6% ± 0.2% using Archimedes’ principle, corresponding to an internal volumetric porosity below 0.4%. The initial average surface roughness (Ra) of the as-built specimens was approximately 8.5 ± 0.8 µm. However, as detailed in the subsequent polishing steps, all substrates were sequentially ground using SiC papers (up to 2000-grit) and polished with alumina suspension down to 0.05 µm to achieve a uniform, mirror-like finish (Ra < 0.05 µm) prior to chemical activation and electrodeposition. This meticulous surface preparation effectively eliminated substrate surface defects and porosity variations, ensuring a uniform current density distribution and strong mechanical interlocking during electrodeposition.

**Figure 1:**
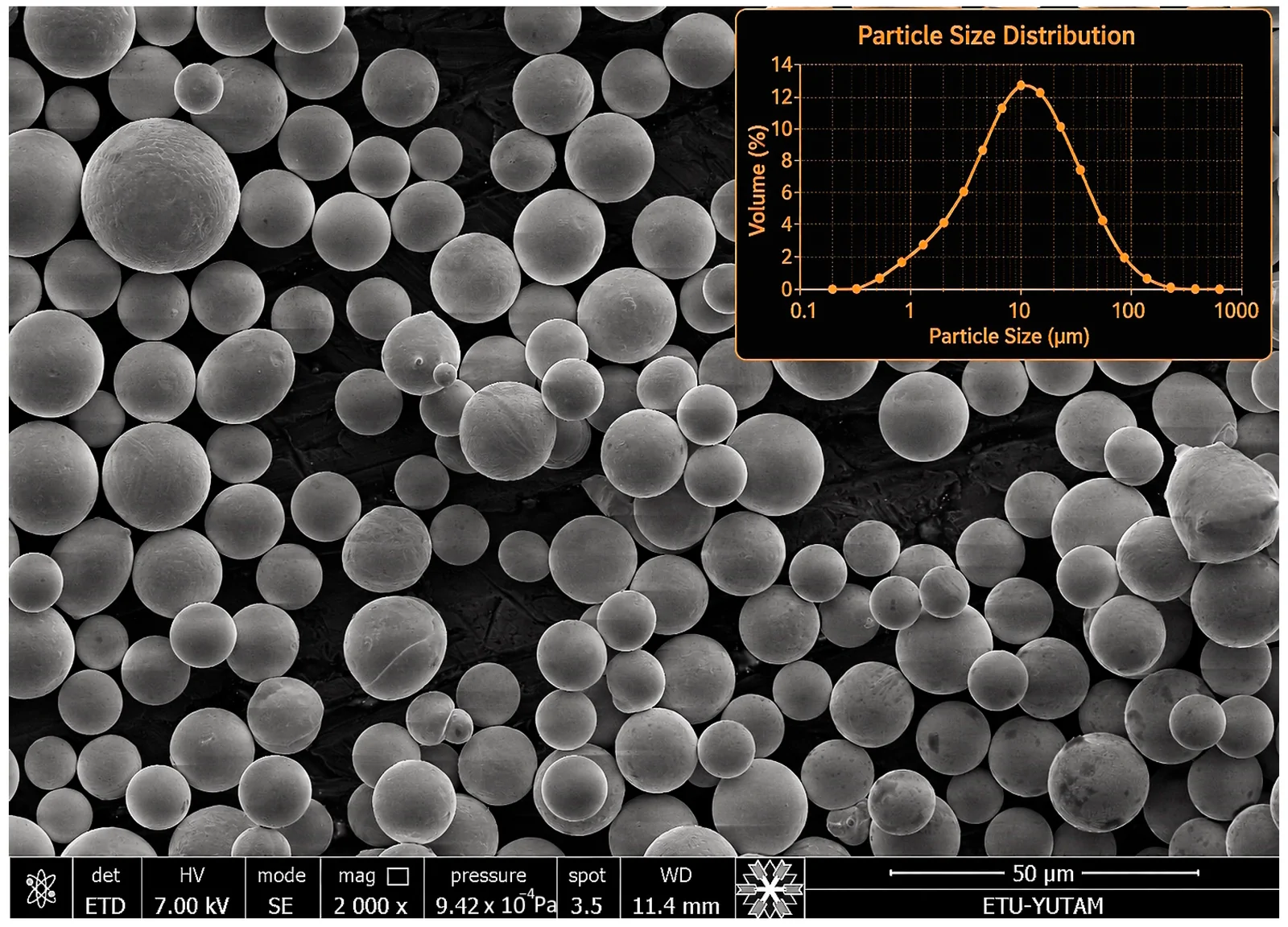
SEM micrograph and image-based particle size distribution of the gas-atomized Ti6Al4V Grade 23 powder used in the present study.

The particle size and size distribution of the gas-atomized Ti alloy powders are presented in Figure 1.

The chemical composition of this powder is given in Table 1. The samples were produced in dimensions of 15 mm × 15 mm × 3 mm. The Ti6Al4V alloy (Grade 23) sample surfaces were used as the cathodic substrates for the electrodeposition process. All samples were subsequently cleaned with ethanol. Prior to deposition, the substrates underwent a multi-step surface preparation process to remove surface oxides, enhance surface activity, and promote adhesion between the deposited layer and the substrate.

The polished substrates were subsequently ultrasonically cleaned in acetone and ethanol for 10 min each to remove any residual contaminants, grease, or polishing debris. Following mechanical polishing, chemical activation was performed to remove the naturally formed passive TiO_2_ layer on the titanium surface. The activation process involved immersion of the samples in an acid mixture composed of 10 vol% hydrofluoric acid (HF), 40 vol% nitric acid (HNO_3_), and 50 vol% deionized water for 20–30 s. This etching treatment effectively dissolved the oxide layer and exposed a clean, active titanium surface suitable for electrochemical deposition. After acid treatment, the substrates were thoroughly rinsed with deionized water and ethanol to neutralize any acid residues, then air-dried at room temperature. To prevent re-passivation of the titanium surface, the activated substrates were immediately transferred into the electrodeposition bath within one hour after activation.

### 2.2. Electrodeposition of the Coatings

The initial stage of deposition was conducted at a relatively low current density (≈1.0 A/dm^2^ for 5 min) to facilitate nucleation and uniform coating adhesion, after which the current density was gradually increased to the desired range (2.5–3.5 A/dm^2^ for 15 min). This pre-treatment procedure was employed to promote uniform nucleation and improve the interfacial integrity of the deposited coating, thereby reducing the likelihood of interfacial discontinuities during subsequent mechanical and electrochemical testing.

### 2.3. Preparation of Zn–Co-Graphene–TiO_2_ Nanocomposite Solutions

The Zn-Co-Graphene-TiO_2_ nanocomposite coatings were electrodeposited from an optimized aqueous electrolyte bath. The electrolyte was prepared using analytical-grade reagents and deionized water. The bath was designed based on previously reported formulations in the literature with suitable modifications to incorporate both graphene nanoplatelets and TiO_2_ nanoparticles. The concentrations of the bath components are expressed in grams per liter (g/L). In the present study, a baseline Zn-Co electrolyte was first prepared, and the coating deposited from this bath was denoted as Zn-Co. Subsequently, graphene was introduced into the Zn-Co electrolyte at a concentration of 0.1 g/L, and the resulting composite coating was labeled as Zn-Co-G. In a separate formulation, TiO_2_ nanoparticles were incorporated into the Zn-Co bath at a concentration of 1.5 g/L, producing a modified electrolyte from which the Zn-Co-Ti coating was obtained. Furthermore, a hybrid composite electrolyte was developed by the simultaneous addition of graphene (0.2 g/L) and TiO_2_ nanoparticles (1.5 g/L) into the Zn-Co solution. The coating deposited from this electrolyte was labeled Zn-Co-GTi. To evaluate the influence of increased reinforcement content, the concentrations of graphene and TiO_2_ nanoparticles were elevated to 0.3 g/L and 2.0 g/L, respectively. The coating synthesized under these conditions was referred to as Zn-Co-GTiH. The designations and concentrations of all prepared coating solutions are given in Table 2.

The graphene nanoplatelets used in this study were supplied by Merck (product code: 900394) with reported to have an average lateral dimension of <2 µm, a density of 0.2–0.4 g/cm^3^ and a thickness of a few nm. The TiO_2_ nanoparticles were obtained from Merck (product code: 637262) with a reported purity of >99.5% and an average particle size of <100 nm. Regarding the dispersion procedure, graphene and TiO_2_ nanoparticles were introduced into the aqueous Zn–Co electrolyte at the concentrations specified in Table 2. The nanomaterials were dispersed using ultrasonication for 30 min at an ultrasonic power of 50 W (30 kHz). Subsequently, the suspension was mechanically stirred at 300 rpm for 30 min to improve the homogeneity of the dispersion. The total electrolyte bath volume was 100 mL, and the distance between the Ti6Al4V cathode and the Pt anode was maintained at approximately 3 cm. During electrodeposition, agitation was maintained at 30 rpm.

Additionally, both the types and quantities of the chemicals used in the preparation of the solutions and deposition parameters are provided in Table 3.

### 2.4. Characterization of Electrodeposited Coatings

Following sample preparation and electrodeposition, the coating morphology, wear behavior, and electrochemical properties were characterized.

#### 2.4.1. Microstructure Morphology of the Coatings

Following sample preparation and electrodeposition, the coating morphology and elemental composition were characterized. A scanning electron microscope (SEM) (FEI-Quanta FEG-250) was used to obtain surface and cross-sectional images of the electrodeposited coatings and to examine their morphological characteristics. Energy-dispersive X-ray spectroscopy (EDS) coupled with the SEM was used to determine the elemental composition and distribution of the coating constituents. The coating thickness was determined from the cross-sectional SEM images by measuring the coating layer at multiple locations along the coating/substrate interface. The mean coating thickness and standard deviation were calculated from these measurements.

The particle morphology and particle size distribution of the gas-atomized Ti6Al4V Grade 23 powder used for substrate fabrication were evaluated from SEM micrographs. The image-based particle size distribution presented in Figure 1 was obtained by analyzing the SEM micrographs using ImageJ software (1.54r version). The particle dimensions were measured from the SEM images, and the resulting particle size distribution is presented in Figure 1. The chemical composition of the Ti6Al4V ELI powder is given in Table 1.

Phase identification of all coated samples was performed using an XRD (GNR Explorer, Agrate Conturbia, Italy) instrument operated at 30 mA and 40 kV. The diffraction peaks were identified according to the ICDD database. Raman spectra of graphene-doped coatings were obtained using a WITec alpha 300 R Micro-Raman spectrometer (WITec Company, Ulm, Germany) with a 532 nm laser source and 0.3 mW laser power. The three-dimensional surface topography of the uncoated and electrodeposited samples was characterized using a 3D optical profilometer (Bruker-Contour GT Nano GmbH, Berlin, Germany). The surface roughness parameter (Ra) was determined from the three-dimensional surface profiles obtained over the analyzed surface areas.

#### 2.4.2. Wear Test Measurement

Wear tests were conducted according to ASTM G133-02 [31] using an Al_2_O_3_ ball with a diameter of 5 mm under dry sliding conditions at room temperature, from which the wear rate (W) and coefficient of friction (COF) were calculated. A normal load of 5 N, a sliding velocity of 10 mm/s, a stroke length of 8 mm, and a test duration of 1200 s were applied. Each test was repeated twice under identical conditions. The wear volume was determined from the three-dimensional wear-track profiles measured using an optical profilometer. The wear rate was calculated using (W = V/(F × L)), where (W) is the wear rate (mm^3^/Nm), (V) is the wear volume (mm^3^), (F) is the applied normal load (N), and (L) is the total sliding distance (m). The experiments were carried out on a Bruker UMT tribometer. Wear test parameters are given in Table 4.

#### 2.4.3. Corrosion and Electrochemical Measurements of the Coatings

Electrochemical measurements were performed using a Gamry Series G^750^ potentiostat/galvanostat in a conventional three-electrode cell. The coated specimen was used as the working electrode, a saturated Ag/AgCl electrode was employed as the reference electrode, and a graphite rod was used as the counter electrode. The exposed working electrode area was 0.502 cm^2^. Prior to potentiodynamic polarization measurements, the specimens were immersed in SBF and the open-circuit potential (OCP) was monitored until stabilization. Additionally, after monitoring the OCP, the specimens were allowed to equilibrate for an additional 300 s. Potentiodynamic polarization measurements were subsequently performed at a scan rate of 0.5 mV s^−1^. The polarization scan was initiated at OCP − 1.5 V and continued in the positive direction to OCP + 1.5 V. For the representative specimen, the corresponding measured potential range was approximately −2.51 to +0.49 V vs. Ag/AgCl. The corrosion potential (Ecorr) and corrosion current density (Icorr) were determined from the polarization curves using the Tafel extrapolation method. All measurements were carried out in Kokubo SBF at 37 °C. The composition of the SBF solution is given in Table 5. Before polarization measurements, the samples were allowed to stabilize at their open-circuit potential (OCP) for a sufficient duration to ensure electrochemical equilibrium. Electrochemical impedance spectroscopy (EIS) measurements were performed at the OCP over a frequency range of 10 mHz to 100 kHz, applying a sinusoidal perturbation with an amplitude of 10 mV. The obtained impedance results were subsequently analyzed using equivalent circuit models to determine the charge transfer resistance and assess the overall corrosion performance.

## 3. Results and Discussion

### 3.1. Microstructural and Compositional Characterization of the Coating

**Figure 2:**
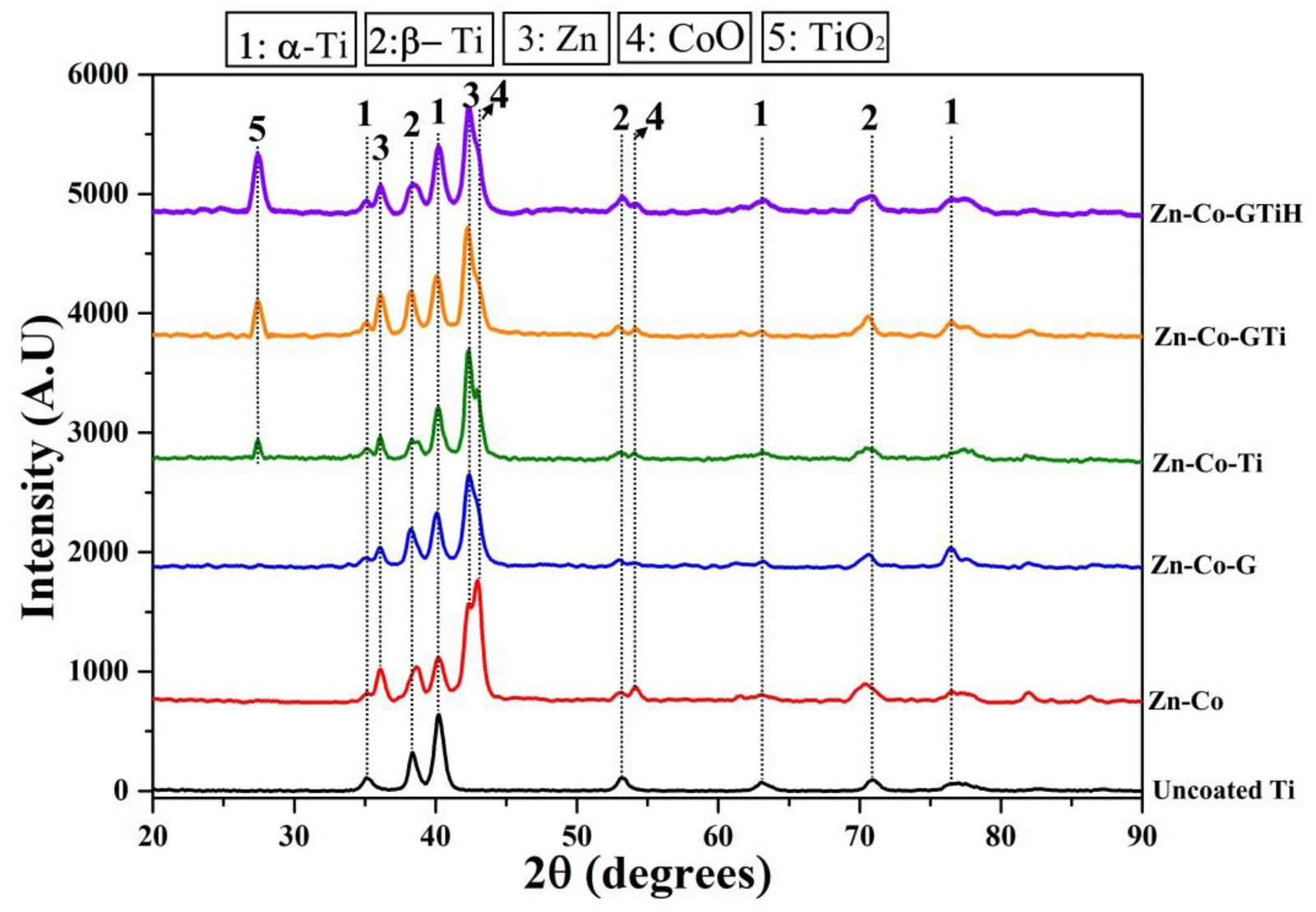
X-ray diffraction patterns of the uncoated and Zn-Co coated samples.

X-ray diffraction (XRD) analysis was conducted to examine the structural properties of the uncoated and electrodeposited samples, and the results are presented in Figure 2.

The diffraction pattern of the untreated substrate displayed only the characteristic reflections of the α-Ti (hcp) and β-Ti (bcc) phases (ICDD card no:00-044-1294). Upon electrodeposition of the Zn-Co-based coatings, a substantial decrease in the intensity of Ti-related peaks was observed, indicating the effective coverage of the substrate surface and the formation of a continuous coating layer. Concurrently, new diffraction peaks corresponding to metallic Zn (ICDD card no:00-004-0831) and Co compounds (ICDD card no:00-048-1719) phases emerged, confirming the successful incorporation of these elements into the alloy matrix during deposition. A detailed examination revealed that the pure Zn-Co coating exhibited sharp and well-defined Zn(101), Zn(002), and Co(111)/(200) reflections, characteristic of a crystalline Zn-Co alloy system. In contrast, the Zn-Co-G sample showed a noticeable broadening and attenuation of these peaks. This reduction in peak sharpness is attributed to the presence of graphene nanosheets, which induce lattice distortion, restrict crystal growth, and promote the formation of a more compact nanocomposite structure [32,33]. As expected, the presence of graphene could not be directly confirmed through XRD due to its weak diffraction intensity and partially amorphous nature; therefore, Raman spectroscopy was employed as a complementary technique to verify graphene incorporation. In the Zn-Co-Ti coating, Zn and Co reflections were still present; however, a pronounced increase in the Co peak intensity was detected. This shift suggests a strong interaction between Co and oxygen species originating from TiO_2_ particles, likely forming Co-O bonds or localized oxide-rich domains during the electrodeposition process. Regarding the TiO_2_ phase, the diffraction feature observed at approximately 27.44° (2θ), marked as TiO_2_ in Figure 2, can be indexed to the rutile TiO_2_ (110) reflection according to ICDD PDF No. 00-021-1276. The additional rutile reflections expected at approximately 36.03° (101), 39.16° (200), 41.12° (111), and 54.27° (211) are located in regions where diffraction features from the Zn-Co coating and the Ti6Al4V substrate are also present; therefore, these reflections cannot be independently resolved with sufficient confidence in the present pattern. Accordingly, the TiO_2_ assignment is based primarily on the clearly observable rutile (110) reflection, while the overlapping features are interpreted cautiously. Finally, in the Zn-Co-GTi and Zn-Co-GTiH coatings, the progressive increase in TiO_2_ content resulted in a marked decrease in the intensity of Zn peaks, which gradually merged with the dominant Co reflections. This behavior indicates that TiO_2_ particles not only refine the overall crystal structure but also modulate the relative phase composition of the coating by enhancing Co dominance within the alloyed matrix.

**Figure 3:**
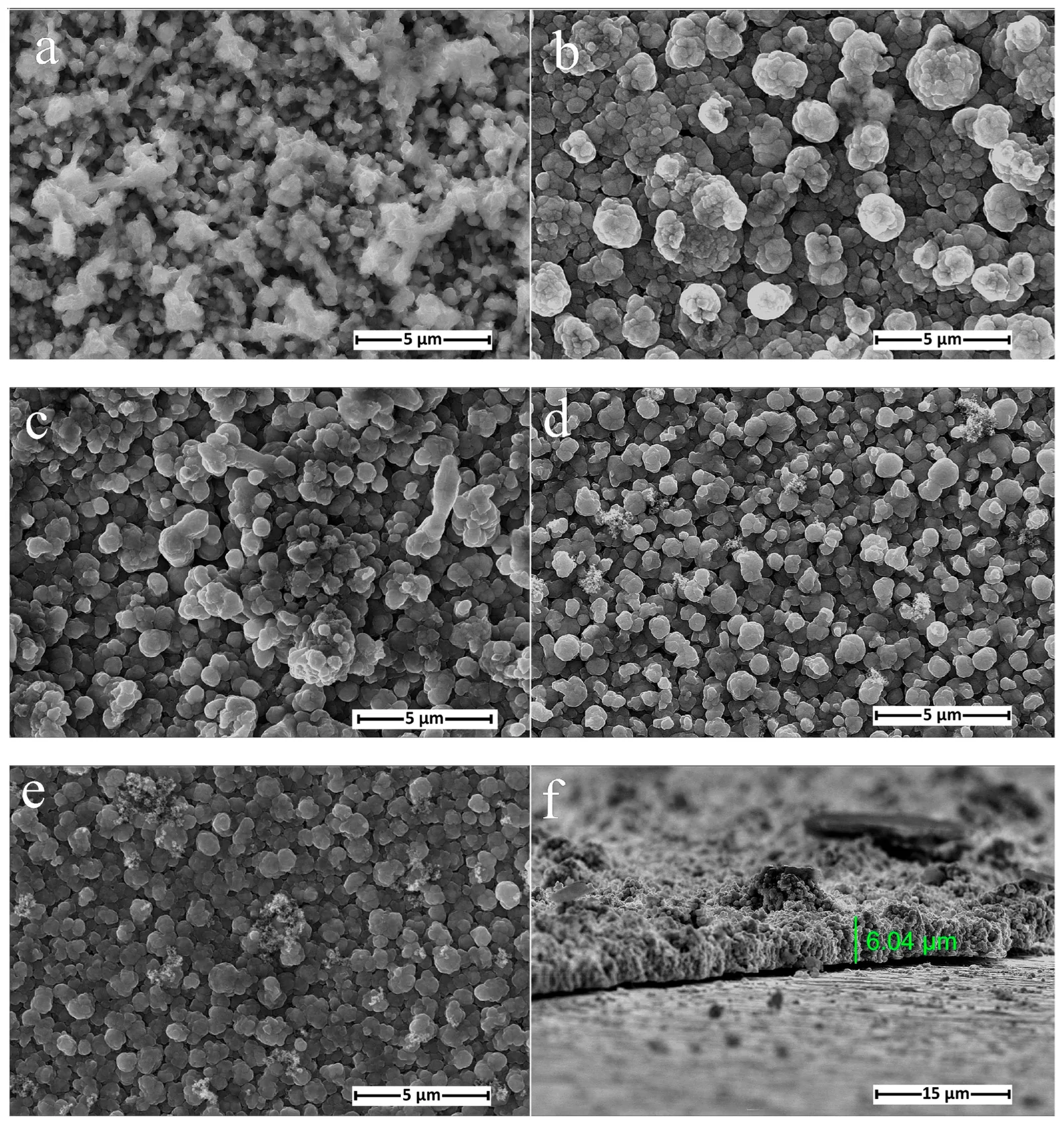
SEM images of (**a**) Zn-Co, (**b**) Zn-Co-G, (**c**) Zn-Co-Ti, (**d**) Zn-Co-GTi, (**e**) Zn-Co-GTiH and (**f**) cross-sectional SEM image of the Zn-Co-GTi coating.

Based on SEM observations, the images shown in Figure 3a–f illustrate the microstructural evolution of Zn-Co-based coatings electrodeposited with different additives and concentrations. The additive-free Zn-Co coating (Figure 3a) exhibits a heterogeneous surface morphology consisting of irregularly distributed and coalesced nanoclusters. The lack of clearly defined grain boundaries together with localized agglomerations indicates that nucleation and grain growth occurred in a largely uncontrolled manner during the deposition process.

Upon the incorporation of graphene (Zn-Co-G) (Figure 3b), a pronounced improvement in surface uniformity was observed a pronounced improvement in surface uniformity, accompanied by a noticeable reduction in grain size and a more compact arrangement of nanoclusters. These features suggest that graphene plays an active role during electrodeposition, most likely by introducing additional nucleation sites that promote grain refinement. The SEM image of the Zn-Co-Ti coating (Figure 3c) reveals a morphology dominated by relatively larger and more rounded grains. This morphological change suggests that TiO_2_ modifies the growth kinetics within the deposition bath, facilitating grain coalescence and leading to the formation of locally compact regions. When graphene and Ti are introduced simultaneously (Figure 3d), the resulting Zn-Co-GTi coating displays a more balanced microstructure in terms of both grain refinement and surface continuity. This behavior suggests a synergistic interaction between the grain-refining effect of graphene and the growth-regulating influence of TiO_2_. In contrast, increasing the Ti content (Zn-Co-GTiH) (Figure 3e) results in the reappearance of larger, locally agglomerated nanoclusters and enhanced surface heterogeneity. These observations suggest that excessive TiO_2_ addition may partially disrupt the homogeneous distribution of graphene within the coating, thereby inducing microstructural irregularities. Overall, the SEM findings obtained in this study demonstrate that graphene incorporation consistently enhances grain refinement and surface homogeneity, whereas Ti addition improves the microstructure only up to an optimal concentration, beyond which its effect becomes detrimental. Figure 3f shows the cross-sectional SEM micrographs of Zn-Co-GTi of the reinforced nanocomposite ceramic coating. Cross-sectional thickness measurements were also performed for the other coating compositions, and no notable differences were observed. The average coating thickness was 6.04 ± 0.5 μm.

**Figure 4:**
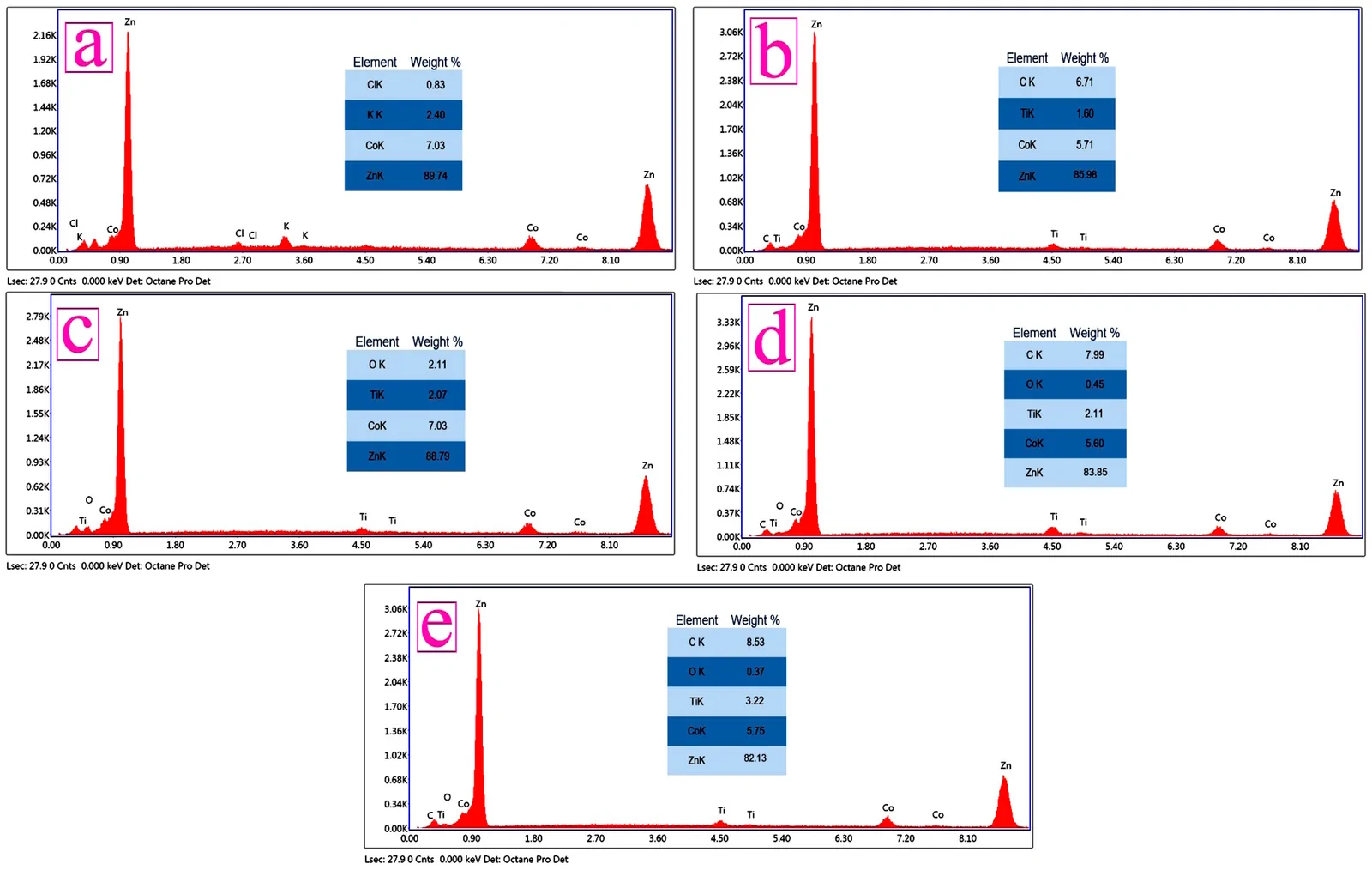
EDS spectra of (**a**) Zn-Co, (**b**) Zn-Co-G, (**c**) Zn-Co-Ti, (**d**) Zn-Co-GTi, and (**e**) Zn-Co-GtiH.

The EDS spectra of the samples coated via electrodeposition are presented in Figure 4. To further verify the incorporation and spatial distribution of the reinforcement phases within the electrodeposited coatings, energy-dispersive X-ray spectroscopy (EDS) analysis was performed. The EDS spectra confirmed the presence of the major elements associated with the Zn-Co matrix and the incorporated reinforcement phases. In the graphene-containing coatings, a distinct carbon signal was detected, while Ti and O signals were observed in the TiO_2_-containing coatings. The corresponding elemental maps further demonstrated the spatial distribution of these elements across the analyzed coating surfaces.

**Figure 5:**
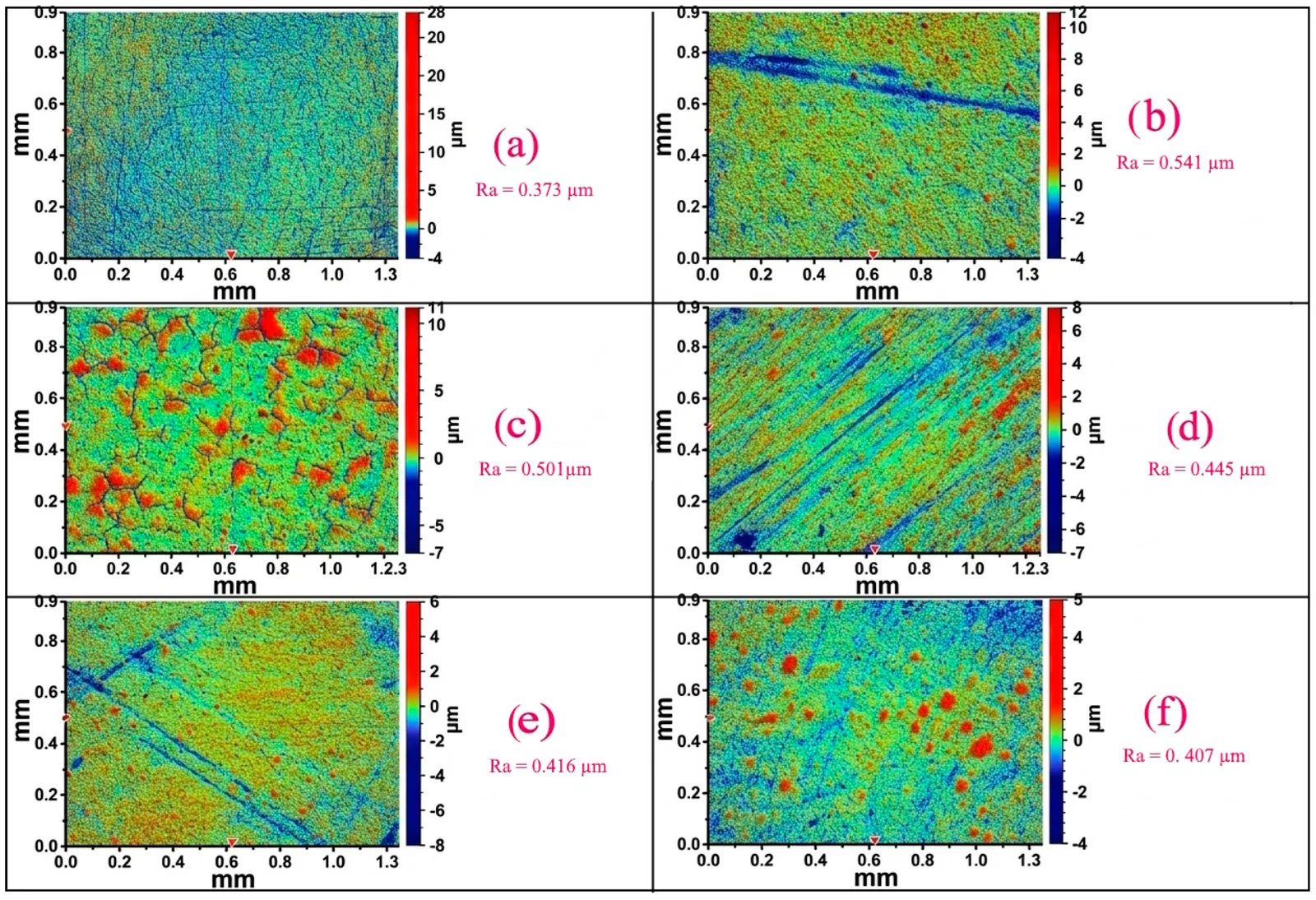
3D Optical profilometer images of worn surfaces: (**a**) uncoated, (**b**) Zn-Co, (**c**) Zn-Co-Ti, (**d**) Zn-Co-G, (**e**) Zn-Co-GTiH, and (**f**) Zn-Co-GTi. The red triangle represents the center of the surface from which the measurement was taken.

The three-dimensional surface topographies and corresponding surface roughness values of the uncoated and coated samples are presented in Figure 5. The measured Ra value of the uncoated Ti6Al4V substrate was 0.373 µm. Following electrodeposition, the Zn-Co coating exhibited an Ra value of 0.541 µm. The incorporation of graphene and TiO_2_ progressively modified the surface topography, with Ra values of 0.501 µm for Zn-Co-Ti, 0.445 µm for Zn-Co-G, 0.416 µm for Zn-Co-GTiH, and 0.407 µm for Zn-Co-GTi. Compared with the unreinforced Zn-Co coating, the addition of graphene reduced the surface roughness, while the incorporation of TiO_2_ produced a further reduction in Ra. The Zn-Co-GTiH coating exhibited an Ra value of 0.416 µm, corresponding to a reduction of approximately 23% relative to the Zn-Co coating. The Zn-Co-GTi coating exhibited the lowest Ra value among the coated samples (0.407 µm), representing an approximately 25% reduction compared with the Zn-Co coating. These profilometric results provide quantitative evidence that the incorporation of graphene and TiO_2_ modifies the surface topography of the electrodeposited coatings. In particular, the reduction in Ra observed for the hybrid coatings is consistent with the more uniform surface morphology observed in the SEM micrographs. However, the roughness results also indicate that the coating surfaces remain rougher than the polished uncoated substrate. Therefore, the effect of the reinforcement phases is interpreted primarily as a modification and homogenization of the coating surface rather than an absolute reduction in roughness relative to the uncoated Ti6Al4V substrate.

**Figure 6:**
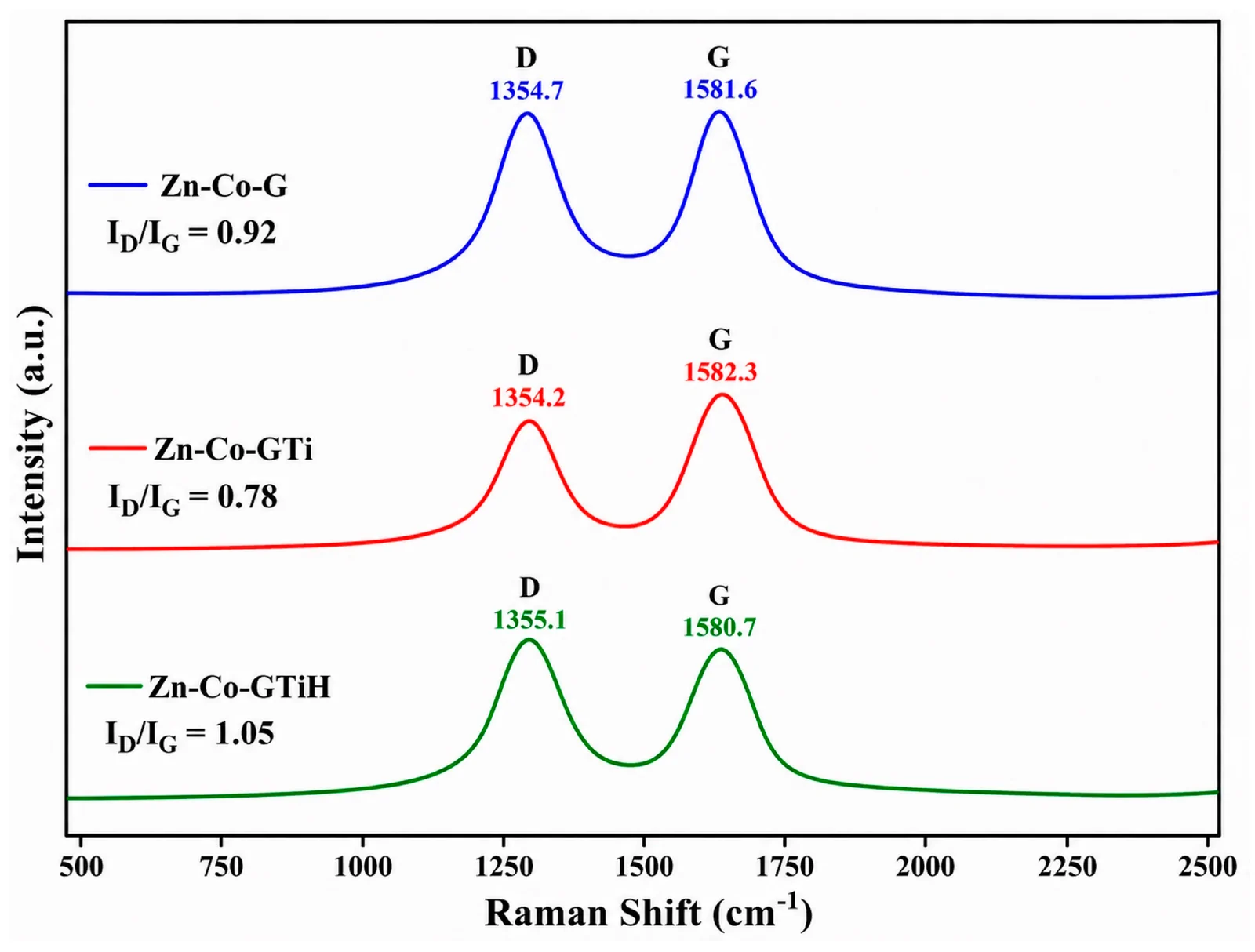
Raman spectra of graphene-containing coatings. The characteristic D and G bands of graphene.

The Raman spectra of Zn-Co-G, Zn-Co-GTi, and Zn-Co-GTiH (Figure 6) exhibit two characteristic bands centered at approximately 1350 cm^−1^ (D band) and 1580 cm^−1^ (G band), corresponding to defect-induced vibrations and the in-plane stretching of sp^2^-hybridized carbon atoms, respectively.

The Raman spectra of Zn-Co-G, Zn-Co-GTi, and Zn-Co-GTiH exhibited the characteristic D and G bands of graphene at approximately 1350 and 1580 cm^−1^, respectively (Figure 6). The D- and G-band positions were determined as 1354.7/1581.6 cm^−1^ for Zn-Co-G, 1354.2/1582.3 cm^−1^ for Zn-Co-GTi, and 1355.1/1580.7 cm^−1^ for Zn-Co-GTiH. The corresponding I_D_/I_G_ ratios were 0.92, 0.78, and 1.05, respectively. The lower I_D_/I_G_ ratio of Zn-Co-GTi indicates a relatively lower degree of structural disorder and a more ordered graphitic structure compared with Zn-Co-G and Zn-Co-GTiH. In contrast, the higher I_D_/I_G_ ratio observed for Zn-Co-GTiH indicates an increased degree of defect-related disorder, which is consistent with the localized agglomeration observed at the higher reinforcement content [34]. These results demonstrate that the modification process alters the defect density without disrupting the carbon framework, which may enhance the functional properties of the material.

### 3.2. Wear Behaviour

**Figure 7:**
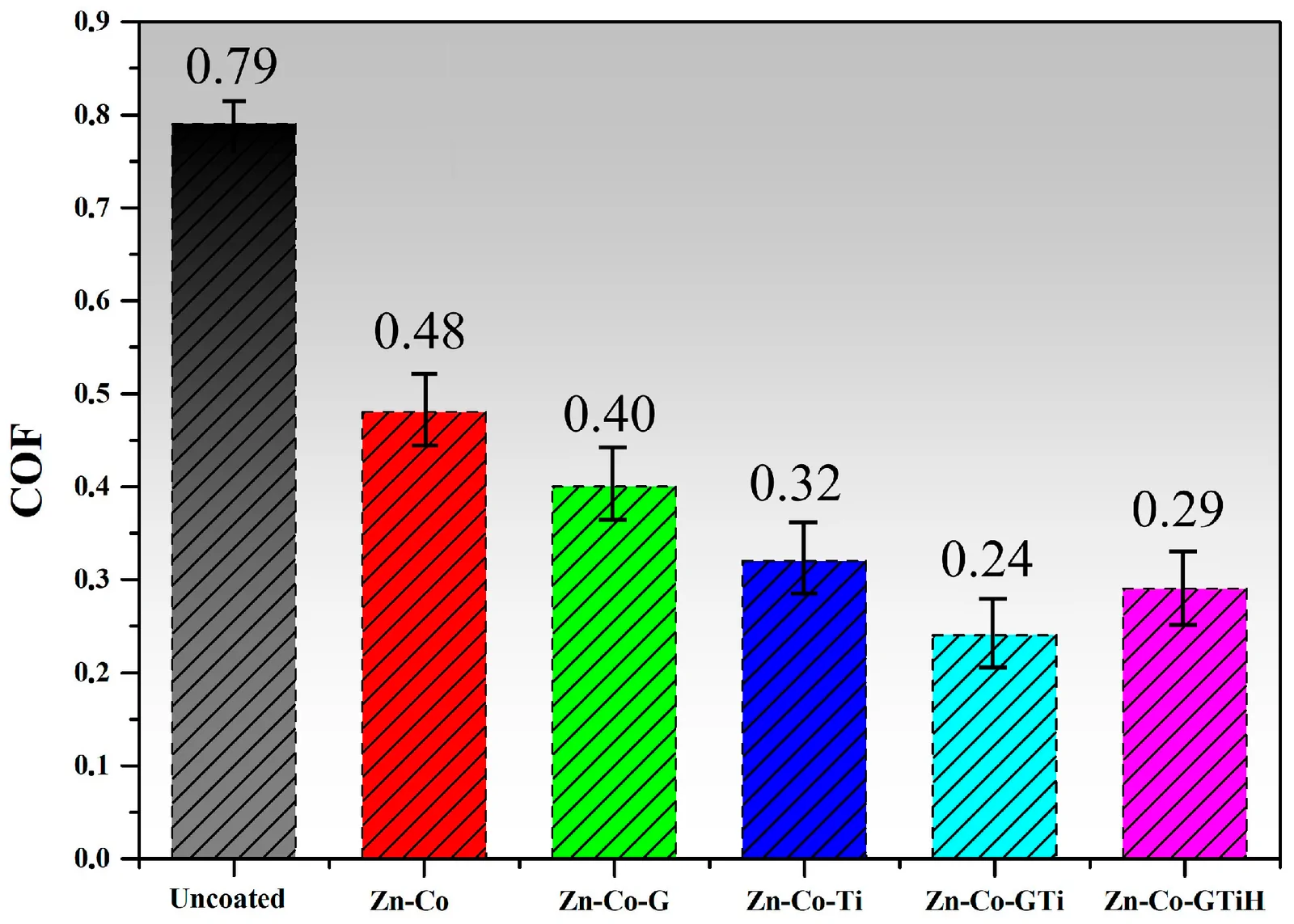
Friction coefficients of the uncoated and Zn-Co coated samples.

Figure 7 illustrates the coefficients of friction of the uncoated substrate, Zn-Co coating, and graphene-TiO_2_ reinforced Zn-Co composite coatings. The uncoated Ti alloy had a coefficient of friction (COF) of 0.79. In contrast, all coated specimens had substantially lower COF values, indicating that the electrodeposited coatings effectively improved the tribological behavior of the substrate.

**Figure 8:**
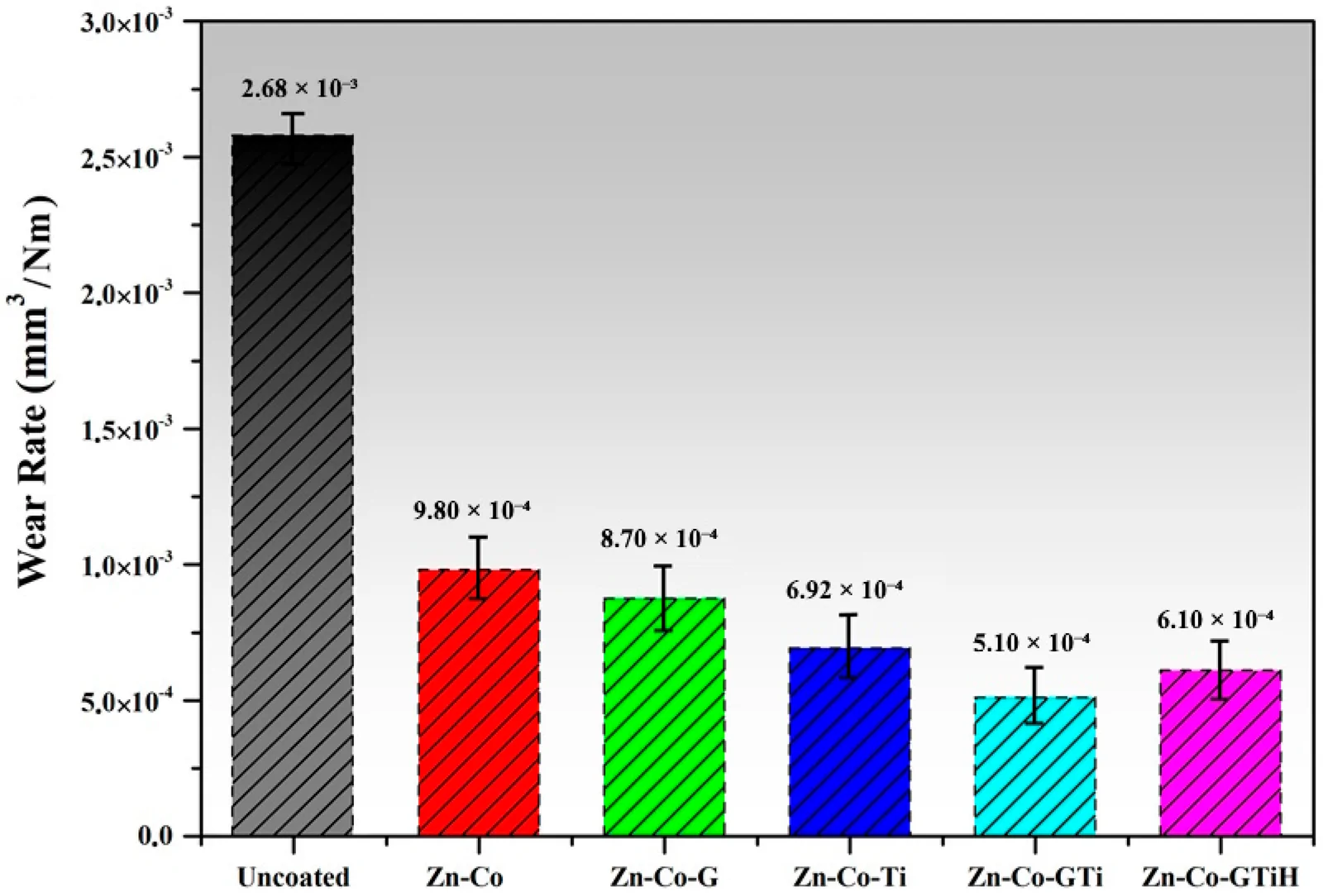
Wear rates of the uncoated and Zn-Co coated samples.

Among the coated samples, the Zn-Co-GTi coating demonstrated the lowest COF (0.24), representing the highest friction-reducing performance. Coatings without graphene or TiO_2_ incorporation exhibited a COF of 0.48, whereas the incorporation of graphene alone reduced the COF to 0.40. Similarly, coatings containing only TiO_2_ exhibited a further reduction in the COF to 0.32. In the coating containing higher loadings of both graphene and TiO_2_ (Zn-Co-GTiH), the COF was measured as 0.29. These findings suggest that the incorporation of graphene and TiO_2_, either individually or in combination, significantly enhances the friction-reducing capability of the coating compared with the unmodified coating, with the Zn-Co-GTi coating exhibiting the most pronounced improvement. This observation is thought to be associated with the ceramic nature of the nanocomposite coatings, which exhibit low shear strength, as well as with the ability of the hard composite ceramic film to reduce the contact area between the mating surfaces [35,36]. Composite coatings containing TiO_2_ as the predominant phase may exhibit solid-lubricant behavior. The inherent self-lubricating properties of the TiO_2_ phase contribute to a reduction in the coefficient of friction [37]. Figure 8 shows wear rates of the uncoated substrate, Zn-Co coating, and graphene-TiO_2_-reinforced Zn-Co composite coatings. The uncoated Ti alloy had a wear rate of 2.68 × 10^−3^ mm^3^/Nm. In contrast, all coated specimens exhibited substantially lower wear rate values, indicating that the electrodeposited coatings effectively improved the tribological behavior of the substrate.

**Figure 9:**
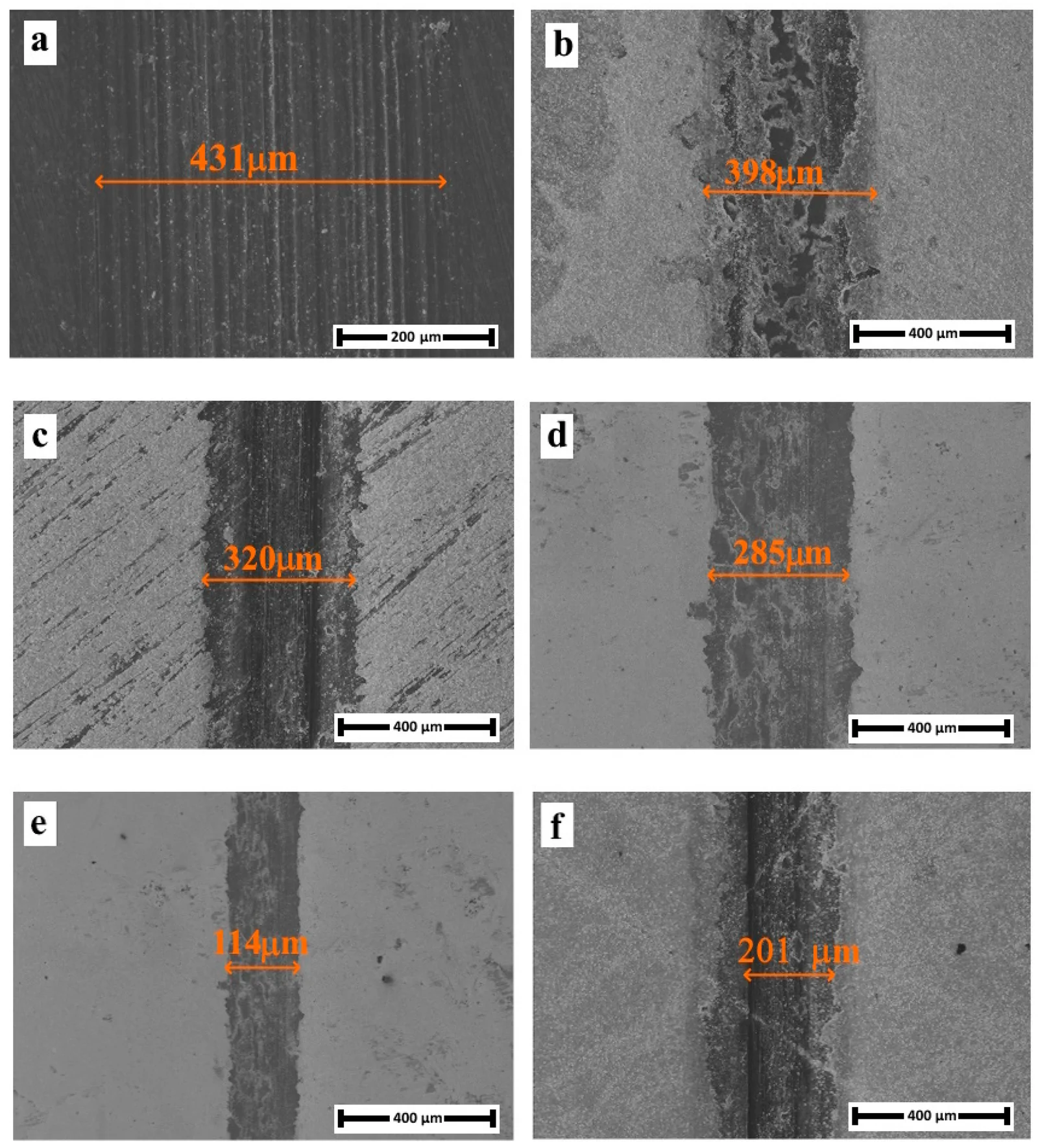
SEM images of worn surfaces: (**a**) uncoated, (**b**) Zn-Co, (**c**) Zn-Co-G, (**d**) Zn-Co-Ti, (**e**) Zn-Co-GTi, and (**f**) Zn-Co-GTiH.

Among the coated samples, the Zn-Co-GTi coating demonstrated the lowest wear rate of 5.1 × 10^−4^ mm^3^/Nm, representing the highest wear resistance performance. Coatings without graphene or TiO_2_ incorporation exhibited a wear rate of 9.8 × 10^−4^ mm^3^/Nm, whereas the incorporation of graphene alone reduced the wear rate to 8.7 × 10^−4^ mm^3^/Nm. Similarly, coatings containing only TiO_2_ exhibited a further reduction in the wear rate to 6.92 × 10^−4^ mm^3^/Nm. In the coating containing higher loadings of both graphene and TiO_2_ (Zn-Co-GTiH), the wear rate was measured as 6.1 × 10^−4^ mm^3^/Nm. The wear rate of the uncoated sample is 2.68 × 10^−3^. These findings suggest that the incorporation of graphene and TiO_2_, either individually or in combination, significantly improves the wear resistance of the coating compared with the unmodified coating, with the Zn-Co-GTi coating exhibiting the most pronounced improvement. The differences in the tribological behavior among the uncoated sample, the Zn-Co coating, and the graphene-TiO_2_-reinforced Zn-Co composite coatings were further investigated by comparing the SEM morphologies of the wear tracks, as presented in Figure 9. Examination of the SEM morphologies of the wear tracks revealed that the uncoated Ti alloy specimen was predominantly subjected to severe adhesive and abrasive wear mechanisms.

The uncoated Ti alloy underwent significant plastic deformation during the wear test due to its relatively low hardness. The resulting deformation increased the real contact area between the pin and the specimen surface, thereby promoting severe adhesive wear. The fine grooves observed within the wear tracks are believed to have been generated by abrasive wear debris formed following the fracture of the naturally occurring oxide film on the surface of the uncoated specimen [38,39]. A comparison of the wear track widths revealed that the widest wear tracks were observed on the uncoated specimens, whereas the narrowest wear tracks were obtained from the specimens coated with the Zn-Co-GTi nanocomposite coating. The superior wear resistance exhibited by the Zn-Co-GTi nanocomposite coating can be attributed to the synergistic effect arising from the incorporation of graphene and TiO_2_ reinforcement phases at their optimum concentrations.

### 3.3. Corrosion Behaviour

**Figure 10:**
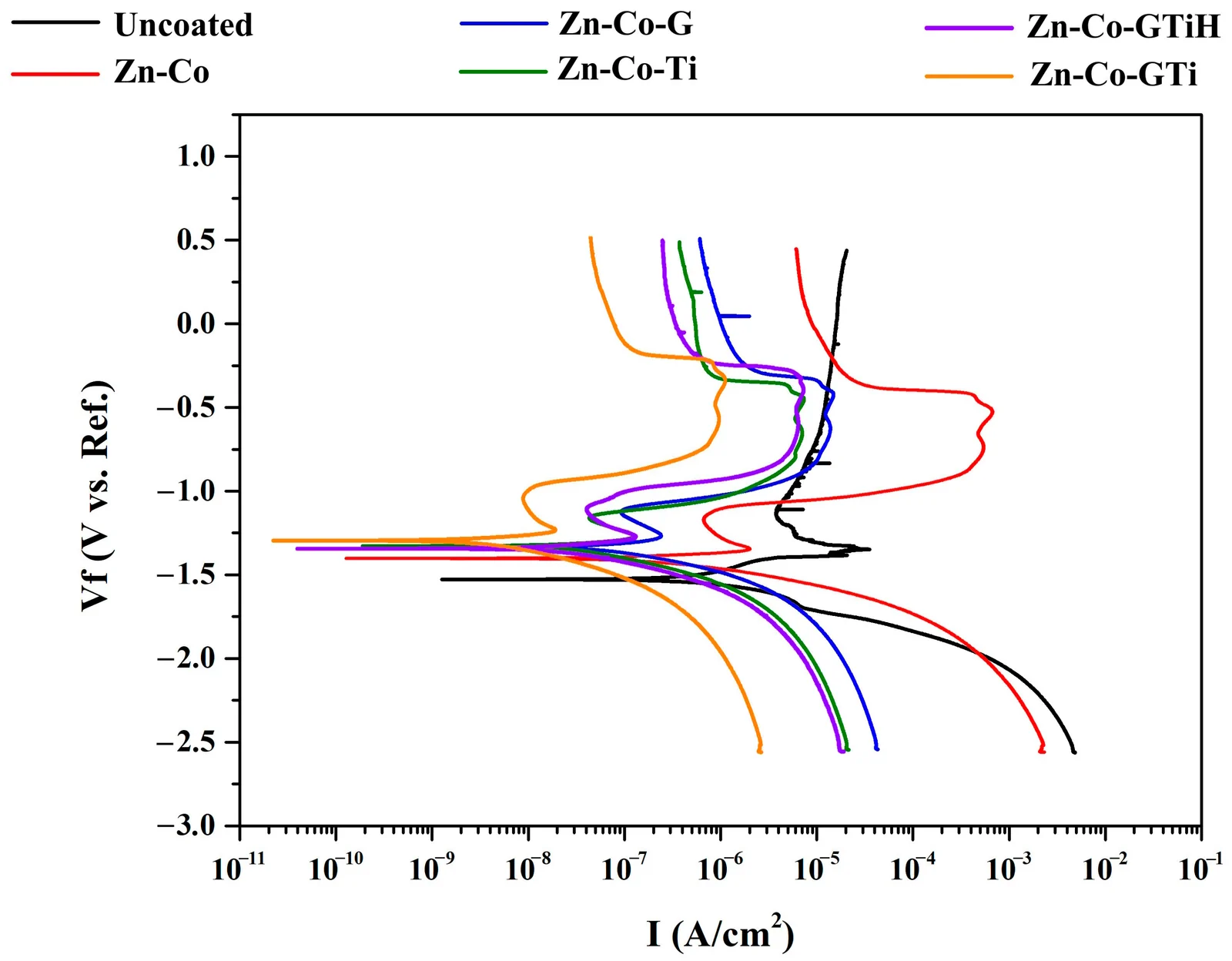
Potentiodynamic polarization curves of the uncoated and Zn-Co coated samples.

Figure 10 illustrates the polarization curves of the uncoated substrate, Zn-Co coating, and graphene-TiO_2_ reinforced Zn-Co composite coatings. Potentiodynamic polarization experiments were conducted by initiating the potential scan from a potential below the open-circuit potential (OCP).

The corrosion potential (E_corr_), corrosion current density (I_corr_), and anodic/cathodic Tafel slopes (β_a_ and β_b_) were determined from the polarization curves using the Tafel extrapolation method, and the resulting corrosion parameters were summarized in Table 6. The polarization resistance (R_p_) of the samples was calculated using the Stern–Geary formula [40].

While the Ecorr value of the uncoated sample was measured as −1553 mV, the corrosion potentials of the Zn-Co, Zn-Co-G, Zn-Co-Ti, Zn-Co-GTi, and Zn-Co-GTiH nanocomposite-coated samples were measured as −1372, −1305, −1312, −1281, and −1297 mV, respectively. A positive increase in corrosion potential was observed in all coated samples compared to the uncoated samples. Another important factor in determining corrosion characteristics is corrosion current (I_corr_). The corrosion current density (Icorr) values of the uncoated, Zn-Co, Zn-Co-G, Zn-Co-Ti, Zn-Co-GTi, and Zn-Co-GTiH samples were measured as 0.914, 0.343, 0.122, 0.101, 0.0059, and 0.091 μA cm^−2^, respectively.

Based on these two results, a noticeable increase in corrosion resistance values was observed in the coated samples [41]. Thus, the addition of Graphene and TiO_2_ to Zn-Co coatings resulted in an increase in the corrosion resistance of the coatings. This improvement can be attributed to the modification of the coating structure resulting from the incorporation of the reinforcing phases. W. Shang et al. [42] demonstrated that composite coatings provide a more effective physical barrier against corrosion than undoped coatings, resulting in improved corrosion resistance. M. Khalil et al. [24] reported that the simultaneous incorporation of TiO_2_ and graphene into Zn-Co coatings can produce a synergistic effect, in which graphene provides a conductive network that facilitates charge transfer, while TiO_2_ contributes to physical barrier strengthening and chemical passivation.

**Figure 11:**
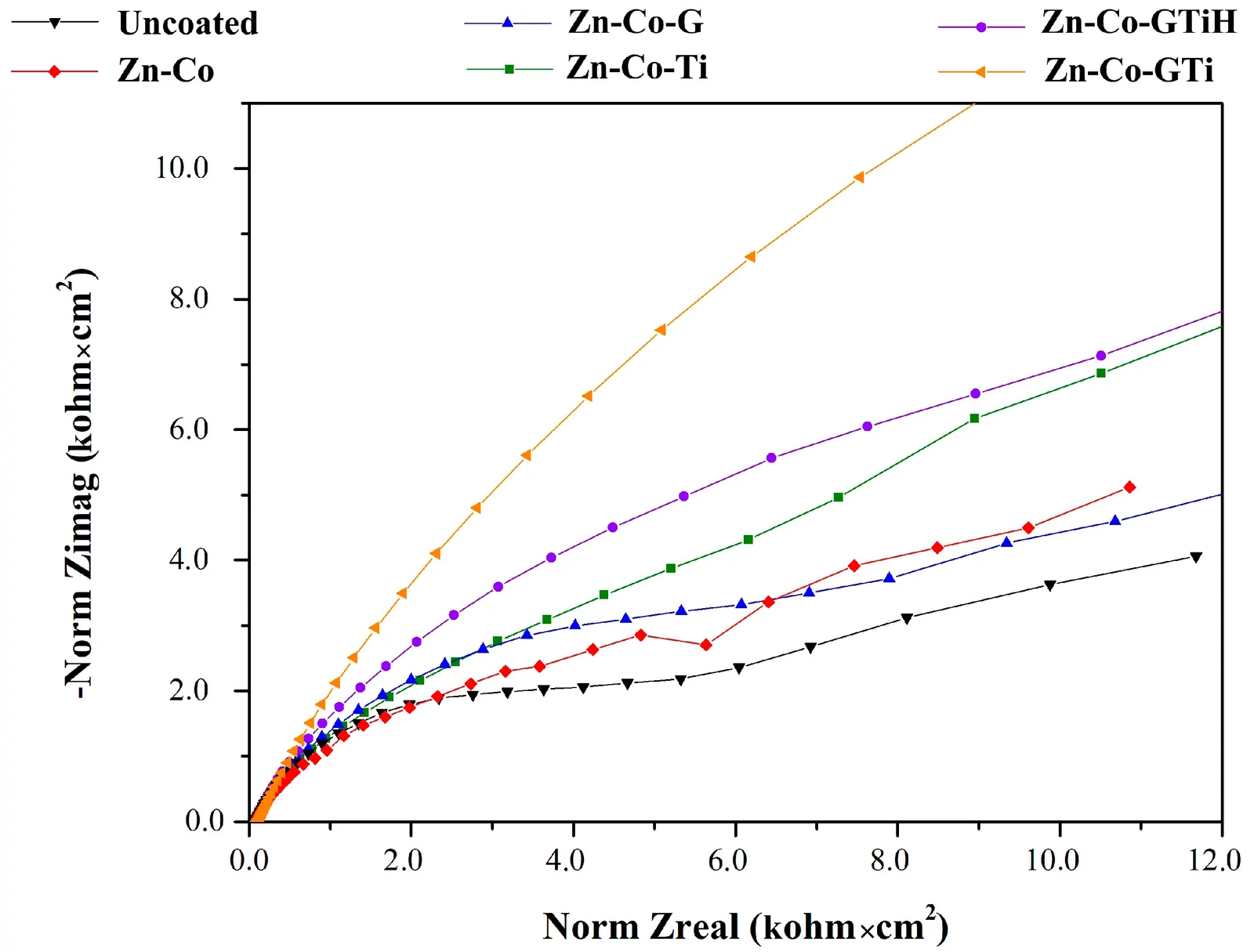
Nyquist plots of the uncoated and Zn-Co coated samples.

**Figure 12:**
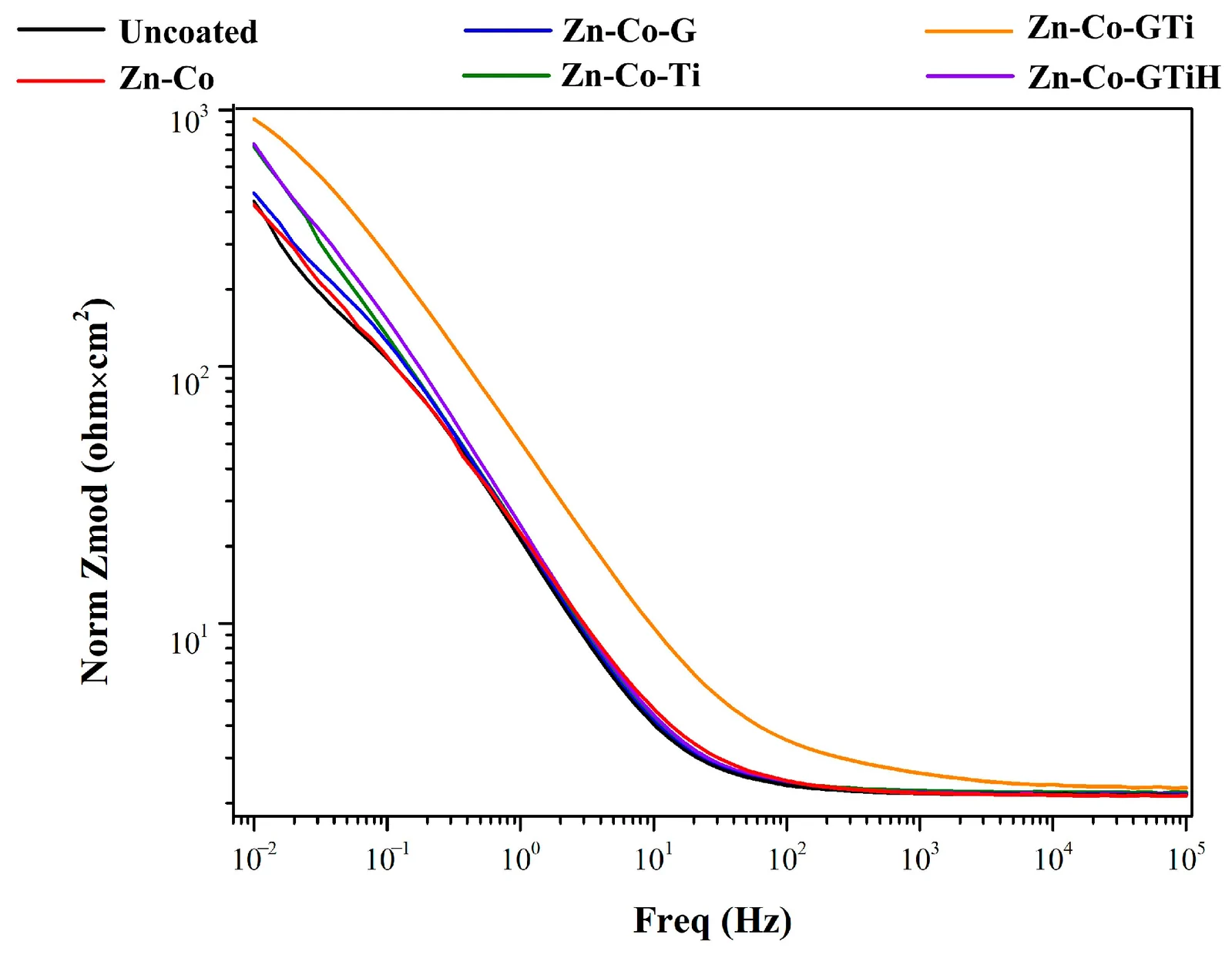
Bode plots of impedance for the uncoated and Zn-Co-coated samples.

**Figure 13:**
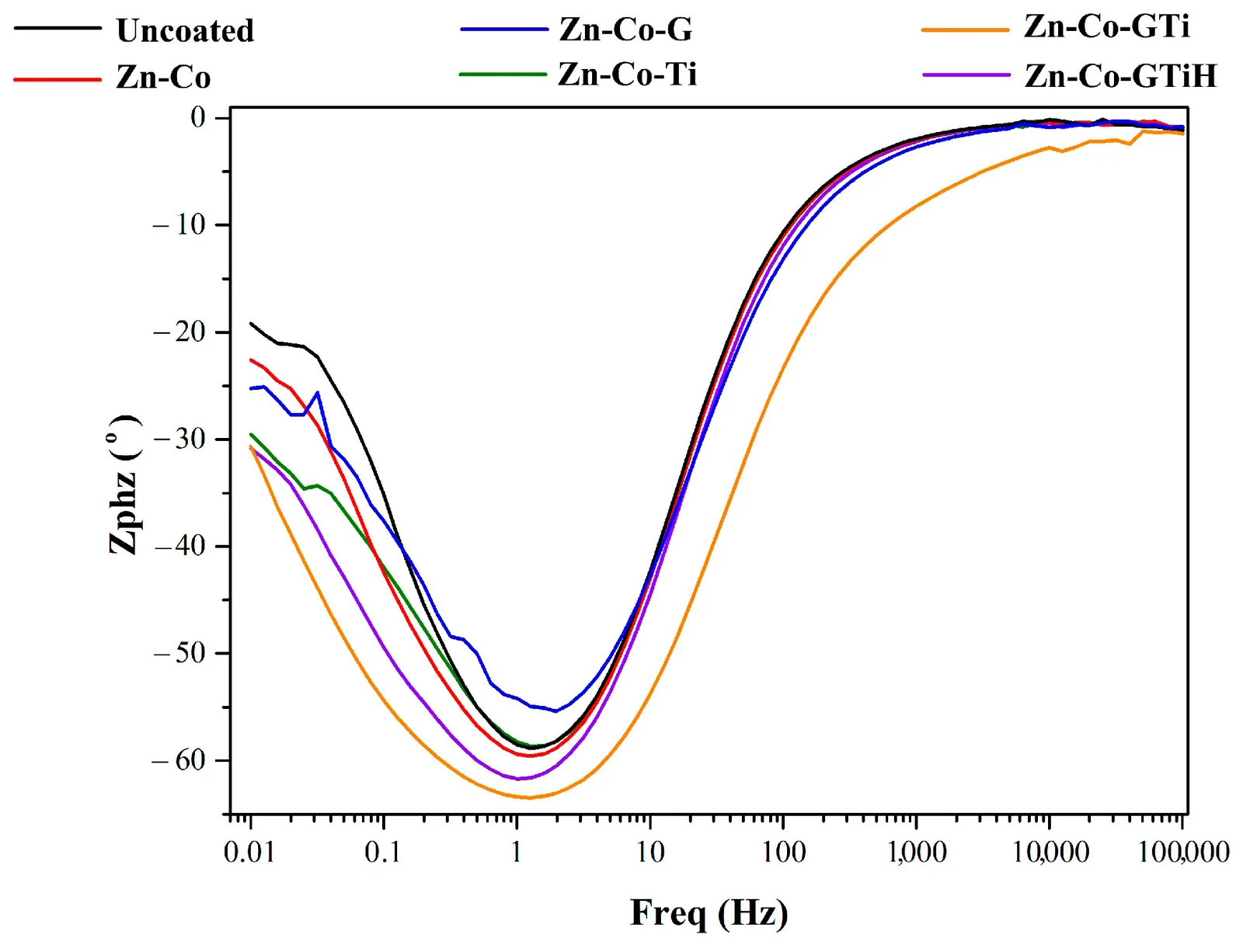
Bode plots of phase angles for the uncoated and Zn-Co-coated samples.

Figure 11, Figure 12 and Figure 13 display the Nyquist and Bode plots of electrochemical impedance spectroscopy (EIS) tests of the uncoated substrate, Zn-Co coating, and graphene-TiO_2_-reinforced Zn-Co composite coatings, respectively. According to the Nyquist graphs (Figure 11), the largest partial capacitive arc was seen in Zn-Co-GTi, while the narrowest partial capacitive arc was observed in the uncoated sample. It is known that when the capacitive arc increases, the corrosion resistance of a sample will increase [43].

The corrosion resistance of the graphene- and TiO_2_-reinforced Zn-Co composite coatings increased with the incorporation of graphene and TiO_2_ into the Zn-Co matrix, as shown in Figure 11. However, increasing the doping ratio resulted in a decrease in corrosion resistance again. To gain a comprehensive understanding of the corrosion mechanism, the Bode impedance and Bode phase angle plots are presented in Figure 12 and Figure 13, respectively. The Bode impedance plots provide further evidence of the differences in electrochemical barrier behavior among the investigated coatings. The Zn-Co-GTi coating exhibited the highest impedance magnitude over almost the entire measured frequency range, particularly at low frequencies, indicating a greater resistance to ionic transport and electrolyte penetration through the coating. The Zn-Co-GTiH coating also showed a relatively high impedance response, although its magnitude was lower than that of Zn-Co-GTi, suggesting that the higher reinforcement content did not provide an additional improvement in the barrier performance. The Zn-Co-G, Zn-Co-Ti, Zn-Co, and uncoated Ti6Al4V samples exhibited considerably lower impedance magnitudes and converged toward similar values at higher frequencies. The enhanced low-frequency impedance of Zn-Co-GTi is consistent with its larger capacitive response observed in the Nyquist plot and indicates improved electrochemical protection of the substrate.

The Bode phase-angle responses further support the differences observed in the impedance magnitude. The Zn-Co-GTi coating exhibited the most negative phase angle, reaching approximately −63° in the low-frequency region, followed by Zn-Co-GTiH at approximately −62°. The other coated and uncoated samples showed lower phase-angle magnitudes, generally approaching −55 to −60°. The more pronounced phase response of Zn-Co-GTi indicates a stronger capacitive contribution and a more effective barrier response at the coating/electrolyte interface. The slightly lower phase response of Zn-Co-GTiH compared with Zn-Co-GTi may be associated with the higher reinforcement content and the possibility of localized particle agglomeration, which can introduce additional pathways for electrolyte penetration. At high frequencies, the phase angles of all samples approached 0°, indicating that the capacitive contribution becomes less dominant in this frequency region.

**Figure 14:**
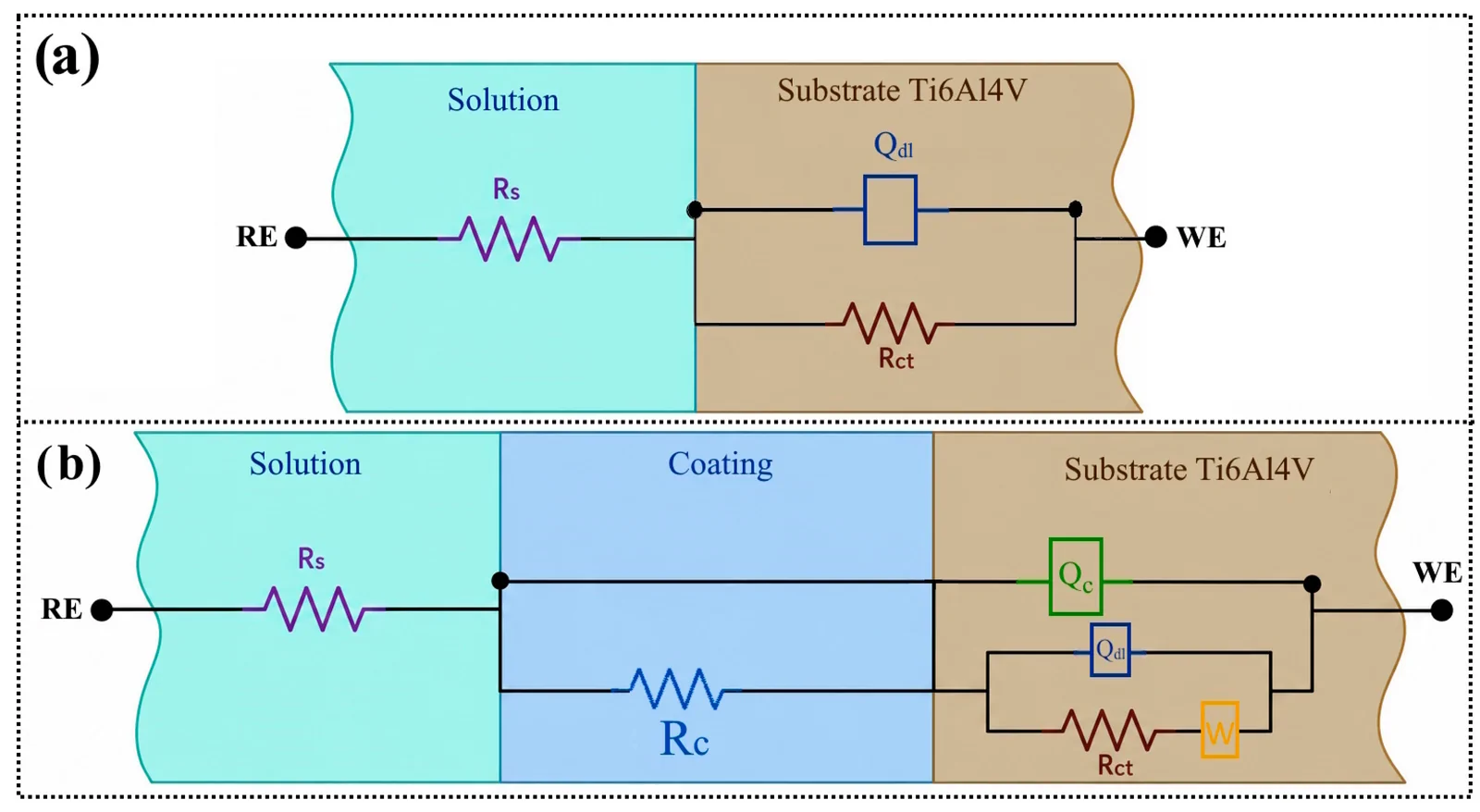
Equivalent circuit for (**a**) uncoated and (**b**) Zn-Co coated samples.

The equivalent electrical circuit models employed to fit the experimental Nyquist and Bode spectra are illustrated in Figure 14. Two different equivalent circuit models were adopted to analyze the electrochemical impedance spectroscopy (EIS) data. The electrochemical behavior of the uncoated specimen was interpreted using the equivalent circuit shown in Figure 14a. In this equivalent circuit, RE is the reference electrode and WE is the work electrode. The electrochemical behavior of the coated specimen was interpreted using the equivalent circuit shown in Figure 14b.

The fitted equivalent circuit obtained for coated samples is displayed in Figure 14b, where Q stands for the constant phase element (CPE) in circuit description code, which is generally used instead of pure capacitance because of the non-ideal character of the corresponding response with phase shifts differing from −90°. The electrochemical impedance spectroscopy (EIS) technique was utilized to define the protective properties of the samples by measuring different parameters, including solution resistance (Rs), charge transfer resistance (Rct), CPE of the double layer (Qdl), coating resistance (Rc) and CPE of the coating (CPEc), and Warburg admittance (W). The excellent agreement between the experimental EIS spectra and the fitted equivalent circuit confirms that the proposed model accurately describes the electrochemical response and corrosion behavior of the investigated specimens. The results obtained from the tests are also given in Table 7.

## 4. Conclusions

Zn-Co, Zn-Co-G, Zn-Co-Ti, Zn-Co-GTi, and Zn-Co-GTiH nanocomposite coatings were successfully electrodeposited onto SLM-fabricated Ti6Al4V substrates. The main findings of this study can be summarized as follows:

- Zn-Co, Zn-Co-G, Zn-Co-Ti, Zn-Co-GTi, and Zn-Co-GtiH nanocomposite coatings were successfully electrodeposited onto SLM-fabricated Ti6Al4V substrates, producing continuous and well-adhered coating layers with an average thickness of approximately 6 μm.
- The incorporation of graphene and TiO_2_ significantly modified the coating microstructure by promoting grain refinement, reducing surface defects, and improving coating compactness. SEM and Raman analyses confirmed the successful incorporation of graphene and demonstrated the structural stability of the carbon framework after electrodeposition.
- The hybrid Zn-Co-GTi coating exhibited the most homogeneous microstructure due to the synergistic interaction between graphene and TiO_2_, whereas excessive nanoparticle addition (Zn-Co-GTiH) resulted in localized agglomeration and partial deterioration of the coating morphology.
- All coated samples showed considerably improved tribological performance compared with the uncoated Ti6Al4V substrate. The Zn-Co-GTi coating achieved the lowest coefficient of friction (0.24) and the lowest wear rate (5.1 × 10^−4^ mm^3^/Nm), indicating superior wear resistance.
- Potentiodynamic polarization measurements demonstrated that the incorporation of graphene and TiO_2_ substantially enhanced corrosion resistance by shifting the corrosion potential toward more noble values while significantly reducing the corrosion current density.
- Electrochemical impedance spectroscopy confirmed the polarization results, with the Zn-Co-GTi coating exhibiting the largest capacitive loop and the highest charge transfer resistance, indicating the formation of an effective protective barrier against electrolyte penetration.
- Overall, the simultaneous incorporation of graphene and TiO_2_ at optimized concentrations produced a clear synergistic effect, leading to superior corrosion and wear resistance compared with single-particle reinforced coatings. These results suggest that Zn-Co-Graphene-TiO_2_ nanocomposite coatings are promising candidates for extending the service life of SLM-fabricated Ti6Al4V components in demanding engineering and biomedical applications.

## Figures and Tables

**Table 1:** Chemical composition of Ti6Al4V ELI alloys (wt.%).

Substrate	Aluminium (Al)	Vanadium (V)	Iron (Fe)	Oxygen (O)	Carbon (C)	Nitrogen (N)	Hydrogen (H)	Titanium (Ti)
Ti6Al4V ELI	5.5–6.5	3.5–4.5	0–0.25	0–0.13	0–0.08	0–0.05	0–0.012	88–91

**Table 2:** Coating labels and their respective compositions.

Samples Names	Graphene (g/L)	TiO_2_ (g/L)
Zn-Co	0	0
Zn-Co-G	0.1	0
Zn-Co-Ti	0	1.5
Zn-Co-GTi	0.2	1.5
Zn-Co-GTiH	0.3	2.0

**Table 3:** Electrodeposition bath composition and deposition parameters.

Component	Chemical Formula	Concentration (g/L)
Zinc source	ZnCl_2_	27.2
Cobalt source	CoCl_2_·6H_2_O	11.9
Supporting electrolyte	Na_2_SO_4_	21.3
Buffer agent	H_3_BO_3_	18.6
Graphene	C (Graphene)	0.1–0.3
TiO_2_ nanoparticles	TiO_2_	1.5–2.0
Surfactant	SDS	0.1
pH	4.5–5.0	
Temperature	45 ± 2 °C	
Current density	1.0–3.5 A/dm^2^	
Deposition time	20 min	
Drying	60 °C for 2 h	
Cathode	Ti6Al4V (activated)	
Anode	Pure Platinum (Pt) Plate	

**Table 4:** Wear test parameters.

Test Parameters	Values
Test type	Reciprocating
Normal load	5 N
Velocity	10 mm/s
Wear stroke	8 mm
Time	1200 s

**Table 5:** Chemical composition of simulated body fluid (SBF).

Order	Reagent	Ion Concentration (mM)
**1**	Na^+^	142
**2**	K^+^	0.5
**3**	Mg^2+^	1.5
**4**	Ca^2+^	2.5
**5**	Cl^−1^	103.0
**6**	HCO_3_^−^	27.0
**7**	HPO_4_^2−^	1.0
**8**	SO_4_^2−^	0.5

**Table 6:** Results of potentiodynamic corrosion tests of uncoated and coated samples in simulated body fluid (SBF).

Samples	*β_a_* (mV/dec)	*β_c_* (mV/dec)	E_corr_ (mV)	I_corr_ (A/cm^2^)	R_p_ (Ω cm^2^)
Uncoated	196.7	90.1	−1553	0.914 × 10^−6^	2.94 × 10^4^
Zn-Co	78.0	111.2	−1372	0.343 × 10^−6^	5.80 × 10^4^
Zn-Co-G	57.3	115.9	−1305	0.122 × 10^−6^	1.36 × 10^5^
Zn-Co-Ti	57.7	104.1	−1312	0.101 × 10^−6^	1.60 × 10^5^
Zn-Co-GTi	71.1	244.4	−1281	0.059 × 10^−6^	4.05 × 10^5^
Zn-Co-GTiH	65.3	155.3	−1297	0.091 × 10^−6^	2.19 × 10^5^

**Table 7:** EIS results for all samples in SBF.

Sample	Rs (Ω)	Rc (Ω)	CPEc (Q)	nc	Rct (Ω)	Qdl	Ndl	W
Uncoated	74.46	3074.87	-	-	-	4.172 × 10^−4^	0.934	-
Zn-Co	84.42	4512.85	3.909 × 10^−4^	0.852	4338.02	2.954 × 10^−4^	0.849	~2.61
Zn-Co-G	85.55	5052.93	3.440 × 10^−4^	0.819	4896.60	2.753 × 10^−4^	0.815	~2.72
Zn-Co-Ti	85.60	5129.04	3.159 × 10^−4^	0.801	5357.91	1.624 × 10^−3^	0.798	~2.91
Zn-Co-GTi	93.37	5950.13	3.077 × 10^−4^	0.753	6111.30	1.556 × 10^−3^	0.721	~3.12
Zn-Co-GTiH	85.86	5516.26	3.256 × 10^−4^	0.788	5905.96	1.715 × 10^−3^	0.748	~3.01

## Data Availability

The original contributions presented in this study are included in the article. Further inquiries can be directed to the corresponding author.
